# Hospital pharmacists understanding of available health literacy assessment tools and their perceived barriers for incorporation in patient education – a survey study

**DOI:** 10.1186/s12913-020-05269-4

**Published:** 2020-05-11

**Authors:** Sara Chan, Sean P. Spina, Dalyce M. Zuk, Karen Dahri

**Affiliations:** 1 Faculty of Pharmaceutical Sciences, University of British Columbia, 2405 Wesbrook Mall, Vancouver, BC V6T 1Z3 Canada; 2 Department of Pharmacy, Royal Jubilee Hospital, Island Health, 1952 Bay St, Victoria, BC V8R 1J8 Canada; 3 Alberta Health Services, 3#308, 3031 Hospital Drive NW, Calgary, AB T2N 2T8 Canada; 4 Vancouver General Hospital, Vancouver Coastal Health, 855 West 12th Avenue, Vancouver, BC V5Z 1M9 Canada

**Keywords:** Health literacy, Pharmacy practice, Medication adherence

## Abstract

**Background:**

Patients with low health literacy experience difficulty in understanding their medications leading to worse health outcomes. Pharmacists need to use formal assessment tools to be able to identify these patients, so they can better tailor their patient education. The objective of the study was to characterize hospital pharmacists understanding of health literacy and their use of screening and counselling strategies before and after completion of an educational module and to identify barriers that hospital pharmacists perceive to exist that prevent them from using health literacy tools.

**Methods:**

Pharmacists in three health authorities were administered a pre-survey and then given access to an online 11 min educational video. The post-survey was distributed 1 month later. Descriptive statistics were used to quantify survey responses with comparisons made between pre and post responses. The main outcome measure was pharmacists’ understanding of health literacy and their current practice related to health literacy.

**Results:**

There were 131 respondents for the pre-survey and 39 for the post-survey. In the pre-module survey, 84% of pharmacists felt they understood what health literacy was, but only 53% currently assessed patients for their health literacy status and 40% were aware of what strategies to use in low health literacy patients. Lack of time (74%) was the biggest barrier in assessing patients’ health literacy. In the post-module survey, 87% felt they understood what health literacy was and 64% incorporated health literacy status evaluation into their clinical practice. The educational module was helpful to the clinical practice of 74% of respondents.

**Conclusion:**

As health literacy can affect a patient’s ability to adhere to their medications it is important for pharmacists to assess this in their patients. While pharmacists self-reported a high degree of understanding of health literacy, they are not regularly assessing their patients’ health literacy status and are unaware of what strategies to use for low literacy patients.

## Background

While literacy refers to an individual’s ability to read and write, the World Health Organization (WHO) defines health literacy to be “the cognitive and social skills which determine the motivation and ability of individuals to gain access to, understand, and use information in ways which promote and maintain good health” [1]. According to WHO, an individual with low health literacy refers to a person who may experience challenges in managing their own health effectively, accessing health services and understanding available health information, thus decreasing the likelihood of making informed health decisions [1]. Approximately 60 % of adults and 88 % of elderly individuals in Canada have low health literacy [2]. Low health literacy has been strongly associated with many negative health outcomes such as greater emergency care use, increased rates of hospitalization, lower use of preventative health services such as mammography screening and yearly flu vaccine and increased risk of mortality [3, 4]. Patients have a decreased ability to demonstrate appropriate self-administration of medications and may misinterpret instructions and have poor adherence to their medication regimens [5, 6]. Previous studies of patients with low health literacy found incorrect medication dosing or administration errors in over 28% of the subjects surveyed with the most frequent mistakes pertaining to dosage measurement and frequency of dosage administration [6]. Rates of low health literacy tend to be higher in individuals who possess at least one of the following demographic characteristics: elderly, ethnic minorities, individuals who have not completed high school, adults who speak a language other than English prior to entering school, and people living in poverty [3, 5].

While these demographic characteristics have been reported, past literature has shown us that it is difficult to accurately identify patients with low health literacy without using standardized assessment methods [7]. In a cross-sectional study of 182 subjects, without formally assessing for health literacy status, medical residents were only able to identify low health literacy in 10 % of the patients, despite 32 % of patients testing positive for low health literacy [8]. This illustrates that the use of a formal assessment tool can greatly help clinicians in accurately identifying individuals with low health literacy [8, 9].

Currently, the most common and widespread screening tools for health literacy are the Rapid Estimate of Adult Literacy in Medicine (REALM) and the Test of Functional Health Literacy in Adults (TOFHLA) [10, 11]. There are shortened and revised versions of each to help promote temporal efficiency in administration of the tools [10, 11]. However, there have been substantial differences noted in gender and ethnicity, despite stratification for education [10, 11]. There has been an increase in the development of new screening tools, particularly instruments that can be administered in under 15 min; however, many of them have not been extensively studied and tested [7, 12–16]. In terms of multi-item screening tools, the Newest Vital Sign (NVS) has been tested multiple times [17–21]. It has shown to be a reliable and accurate measure of health literacy [17, 22, 23]. It takes approximately 3 min to administer [24]. Furthermore, studies reported that patients generally felt satisfied with the administration of the NVS and they did not feel shame for being screened with the tool [25, 26]. However, it does appear to have limited practical use in the elderly African-American adult population [18]. In terms of single-item screening tools, the question of “how confident are you in filling medical forms by yourself?” has shown to be accurate in detecting limited and marginal health literacy [7, 12, 13, 27]. The question can be answered with one of the following options: to a great extent, somewhat, very little, and not at all [7, 12, 13, 27].

Given the variety of the assessment tools available, pharmacists could benefit from learning at least one of these assessment tools in order to more effectively identify patients with low health literacy. Unfortunately, uptake amongst community pharmacists has been low. Studies conducted in community pharmacies have shown that only 7 % of pharmacies reported that they attempted to identify patients with health literacy needs [28]. If health professional driven assessment is not done it is unlikely that patients will self-declare their health literacy needs [29]. Many patients with low health literacy often feel a sense of shame which may discourage them from revealing their health literacy status [29]. One study showed that 67.2% of patients with low health literacy had never told their spouses and 53.4% had never told their children of their difficulties in reading [29].

Pharmacists can utilize different strategies to tailor patient education for individuals with low health literacy. Communication techniques and patient counselling strategies exist to help support the learning of patients with inadequate health literacy [3, 8, 30–33]. However, in order to utilize these strategies, pharmacists need to be able to effectively identify these patients. Hence, the need for pharmacists to integrate the use of health literacy assessment tools into their practice. It is unclear whether hospital-based pharmacists currently assess patients’ health literacy or utilize health literacy screening tools. The objective of this study is to survey pharmacists in hospital-based practices to characterise their understanding of health literacy and their use of screening and counselling strategies prior to and following the completion of an educational module regarding health literacy. In addition, to identify barriers that pharmacists perceive to exist that prevent them from using health literacy tools.

## Methods

Pharmacists in three health authorities [Lower Mainland Pharmacy Services (LMPS), Vancouver Island Health Authority (VIHA), Alberta Health Services (AHS)] were recruited to participate in the study. Ethics approval was obtained from the University of British Columbia (UBC) Clinical Research Ethics Board. All participants gave their informed consent.

A pre and post survey was developed and was based on a comprehensive search of the literature [9, 34]. The pre-survey questions were focused on the pharmacists understanding of health literacy, whether they currently employ any strategies to assess and counsel patients, and identification of the barriers that prevent them from assessing patients’ health literacy. The post-survey assessed the pharmacist’s perspective on the impact of the health literacy module on their practice and whether it influenced their use of screening tools and counselling strategies. It also explored methods to encourage increased use of such strategies. Both sets of survey questions were reviewed by a small group of practicing Canadian pharmacists to ensure face and content validity. The survey questions were not statistically validated. All survey questions were in English only. Surveys were created in FluidSurveys© which at the time of this study was the designated survey tool available through UBC and was in compliance with the privacy rules in British Columbia with all data being stored and backed up in Canada.

The educational module itself was an 11-min educational video titled “Reading Into the Health Literacy of Our Patients”. The investigators created it for the purpose of the study and its content was based on an extensive review of the literature [3–8, 17, 18, 22, 25–27, 30–33, 35, 36]. It focused on educating the viewer on the definition of health literacy, how to assess patients that had low health literacy and the strategies that could be used when counselling patients with low health literacy. Once participants completed the pre-module survey they were given access to the video link in Vimeo©. The video link was also made available to staff outside the context of the study.

### Survey administration

The initial pre-module survey was distributed via group e-mail lists to pharmacists within each of the three health authorities. A reminder e-mail was sent 1 week later. After completion of the initial survey, participants were asked for permission for the study investigators to contact them 1 month later with the post-module survey. The survey was estimated to take approximately 5 min to complete. All of the survey invitations and reminders contained consent information. Consent was implied by responding to the survey. Responses to the survey as a whole and to the individual survey questions was entirely voluntary.

Pharmacists were included in the study if they were involved in any direct patient care activities. Questions were embedded into the start of the survey to screen for this inclusion criteria. An incentive of a draw for one of four $25 coffee gift cards was offered to all potential participants. Funding for the study was from the unrestricted research start-up grant of the primary investigator (KD).

Data analysis consisted of simple descriptive statistics which included total counts, percentages, averages, and standard deviations. Comparisons of responses before and after completion of the educational module was planned. Qualitative analysis of open-ended responses was also done.

## Results

One hundred and thirty-one pharmacists completed the pre-survey and thirty-nine completed the post-survey. Table 1 outlines the baseline demographics of the pre-survey cohort. The overall response rate for the pre-survey was 6% and for the post-survey was 29% (38/131 pre-survey respondents).

**Table 1:** Baseline Characteristics for Participants in Each Health Authority

Health Authority	Number of Participants (Number)	Total Number of Pharmacists (Number)	Overall Response Rate per Health Authority (Percent)	Average Years of Experience (Mean, SD)
LMPS	20	534	4	12.8, 9.7
VIHA	15	186	8	17.5, 11.8
Alberta	96	1461	7	8.5, 5.3 [Note: 33 pharmacists reported > 20 years and were not included in calculation of mean]

Prior to completing the educational module, 84% of the participants felt they understood health literacy with 53% currently assessing patients for health literacy in their practice (Table 2). The most commonly used strategies of assessing a patient’s health literacy were those that relied on subjective observations (Table 3). Patient’s verbal communication (76%), demonstrated understanding of medications (76%) and personal intuition (73%) were the most common assessment methods that pharmacists relied on. During counselling sessions, pharmacists would most often present essential information by itself and use simpler language (81%) when counselling patients with inadequate health literacy. Time constraints were the most common barrier that prevented practitioners from assessing their patients and using counselling strategies.

**Table 2:** Pre-module Survey Questions and Responses – Total (Percent)

	Yes	No	Total
I feel I understand what health literacy is	109 (84)	21 (16)	130
I currently assess patients for their health literacy status as part of my clinical practice.	69 (53)	62 (47)	131
I am aware of what strategies to use when interacting with patients with inadequate health literacy.	53 (41)	77 (59)	130
I am comfortable when counselling patients with inadequate health literacy.	88 (68)	42 (32)	130
Do you use any particular strategies for counselling when interacting with patients with inadequate versus adequate health literacy?	79 (63)	46 (37)	125

**Table 3:** Pre-Module Survey Questions and Responses (*N* = 131)

Question	Responses	Count	Percent
How do you currently assess patients for health literacy?	Personal intuition	95	73
	Patient’s level of formal education attained	37	28
	Patient’s verbal communication	100	76
	Patient’s written communication skills, if observable	35	27
	Patient’s demonstrated use of medications, if observable	73	56
	Patient’s demonstrated understanding of medications, if observable	100	76
	Formal health literacy assessment tool	2	2
	I do not assess patients for health literacy	14	11
	Other	4	3
Which of the following strategies do you use when counselling patients with inadequate health literacy?	Asking the patient to demonstrate what you just taught them and to ask questions	100	76
	Engaging in frequent short appointments, instead of one long appointment	40	30
	Presenting essential information by itself or first, and using simpler language	106	81
	Encouraging the presence of a family member	93	71
	Ensuring that printed materials are easy to read for patients	78	60
	Using more than one source of media in teaching (for example diagrams and video)	28	21
	I do not use any particular strategies when counseling patients with inadequate health literacy	8	6
	Other	9	7
Of the following, which barriers do you feel may deter you from assessing patients for their health literacy?	Lack of financial incentives	3	2
	Time constraints	97	74
	Being liable for results of the assessment	9	7
	Patient's expectations of your clinical practice and your own expectations of your routine in your practice	34	26
	Opinions of your practice leader(s)	2	2
	Feeling insufficiently trained to use assessment tools	80	61
	Other	3	2
Of the following, which barriers do you feel may deter you from using the counselling strategies available?	Lack of financial incentives	5	4
	Time constraints	106	81
	Being liable for results of the assessment	19	15
	Patient’s expectations of your clinical practice and your own expectations of your routine in your practice	19	15
	Opinions of your practice leader(s)	4	3
	Feeling insufficiently trained to use assessment tools	56	43
	Other	13	10
Of the following, which would encourage you to use any of the counselling strategies for patients with inadequate health literacy?	Engaging in interactive small group meetings with your colleagues to discuss the counselling strategies	66	50
	E-mail reminders to use the counselling strategies	18	14
	Computerized decision support to use the counselling strategies	57	44
	Mass media campaign on using the counselling strategies	19	15
	Financial incentives	20	15
	Other	25	19

Of the 38 pharmacists that completed the post-survey, 74% felt that completing the educational module on health literacy was helpful to their clinical practice (Table 4). Time constraints continued to be the biggest challenge to implementing health literacy assessment (72%) and counselling strategies (67%) into their practice (Table 5).

**Table 4:** Post-module Survey Questions and Responses – Total (Percent)

	Yes	No	Total
I feel I understand what health literacy is	34 (87)	4(10)	38
I currently assess patients for their health literacy status as part of my clinical practice.	25 (64)	13 (33)	38
I feel I assess the health literacy status of patients more effectively	21 (54)	17 (44)	38
I feel I use more counselling strategies tailored for patients with inadequate health literacy	21 (54)	17 (44)	38
I feel that completing an educational module on health literacy was helpful for my clinical practice	29 (74)	9 (23)	38

**Table 5:** Post-module Survey Questions and Responses (*N* = 38)

Question	Responses	Total	%
Of the following, which barriers do you feel may deter you from assessing patients for their health literacy?	Lack of financial incentives	2	5
	Time constraints	28	72
	Being liable for results of the assessment	1	3
	Patient’s expectations of your clinical practice and your own expectations of your routine in your practice	14	36
	Opinions of your practice leader(s)	2	5
	Feeling insufficiently trained to use assessment tools	16	41
	Other	5	13
Of the following, which barriers do you feel may deter you from using the counselling strategies available?	Lack of financial incentives	2	5
	Time constraints	26	67
	Being liable for results of the assessment	10	26
	Patient’s expectations of your clinical practice and your own expectations of your routine in your practice	12	31
	Opinions of your practice leader(s)	5	13
	Feeling insufficiently trained to use assessment tools	56	43
	Other	13	10
Of the following, which would encourage you to use any of the counselling strategies for patients with inadequate health literacy?	Engaging in interactive small group meetings with your colleagues to discuss the counselling strategies	15	39
	E-mail reminders to use the counselling strategies	11	28
	Computerized decision support to use the counselling strategies	14	36
	Mass media campaign on using the counselling strategies	5	13
	Financial incentives	6	15
	Other	5	13

Comparisons between pre and post module survey responses to the questions regarding whether participants understand what health literacy is and if they currently assess patients for their health literacy status were not statistically significantly different.

Open-ended responses were also incorporated into the survey design. Qualitative analysis of these responses with categorization of themes found that many of these responses would fit into an existing response. For example, an open-ended response to the pre-survey question of counselling strategies used for patients with health literacy was “Highlighting and writing information in simpler words on teaching sheets.” Which would fit into the existing option of “Ensuring that printed materials area easy to read for patients”.

## Discussion

Our study assessed the pharmacists baseline knowledge of health literacy; their use of health literacy assessment tools and counselling strategies; and whether an educational video intervention had an impact on their practice. In addition, barriers to the implementation of health literacy patient assessment and counselling strategies that prevent pharmacists from integrating these interventions into their clinical practice were also identified. While pharmacists self-reported a high rate of understanding of health literacy, just over half of the study population indicated that they currently assessed patients for health literacy and only 41% were aware of what strategies to use with most relying on just subjective patient observation.

Interestingly while survey respondents were not aware of the strategies to use, 63% indicated they use specific strategies when counselling patients with low health literacy. The majority of the study population seemed to use strategies such as presenting essential information first, asking patients to demonstrate what has been taught and encouraging the presence of a family member. While these strategies have been shown to improve patient comprehension, other standardized communication techniques such as the Indian Health Service Model, the Teach-back method, and the Ask-Me-3 method have been shown to better promote understanding in patients with inadequate health literacy [8, 30, 31, 33]. Multiple resources also exist to provide guidance on patient interaction and also on how to structure teaching information all of which would be valuable to pharmacists to further advance their skills [3, 31].

Time continues to be a barrier for using an assessment tool to screen for patient’s health literacy and in deters pharmacists from using the available counselling strategies as found again in our study. While time constraints are consistently reported as a barrier Welch et al. evaluated the implementation of the Newest Vital Sign (NVS) instrument in a primary care clinic and found that it took 30 s to hand out the forms and instruct patients on how to fill them out [24]. It took an additional 2 min to score and enter the results into the patient’s electronic medical record [24]. Overall, tests such as REALM, S-TOFHLA, and METER take on average 2–12 min and are more accurate than using practitioner self-assessment [13]. Patients can also be simply asked questions as a single-item screening tool [7, 12, 13, 27]. For example, the question of “how confident are you in filling medical forms by yourself?” with patients being given the following options as answers to the question: to a great extent, somewhat, very little, and not at all has shown to be accurate in detecting limited and marginal health literacy [7, 12, 13, 27]. Or pharmacists can adopt Health Literacy Universal Precautions as recommended by the Agency for Healthcare Research and Quality where strategies used to educate low health literacy patients are done for everyone [37].

Since the response rate for the post-survey was considerably lower than the pre-survey, we were not able to assess whether the educational intervention had an impact on practice. Other studies that have looked at factors that influence the adoption and implementation of health literacy tools in community pharmacy settings have identified a number of barriers that prevent implementation. Factors preventing uptake include limited support from leadership, higher prioritization of other activities, lack of qualified staff and complexity of the tool being used [38]. The second most reported barrier in our study to implementation of health literacy strategies was a feeling of being insufficiently trained to use the assessment tools. While we were not able to draw conclusions from the educational intervention that we implemented, a prior study that assessed the implementation of the Agency for Healthcare Research and Quality pharmacy health literacy assessment tool in a community pharmacy setting found that the training program had limited impact on patient and staff responses [39].

There were many limitations of this study. We did not prospectively assess pharmacists understanding of health literacy; we asked them to self-report if they felt that they had an understanding. The poor response rate for the post-survey limits our ability to determine if the educational intervention had any impact on the pharmacists’ practice. While we educated the pharmacists on what tools were available we did not provide any detailed training on how to use the tools. In addition, our educational intervention was limited to a short video.

## Conclusion

Our study did highlight a need for organized educational interventions to pharmacists on health literacy. Pharmacists may self-report a good understanding of health literacy, but they do not regularly assess their patients’ health literacy status and are unaware of what strategies should be used for low literacy patients. From our survey, pharmacists indicated that they would prefer engaging in interactive small group meetings with colleagues or utilizing computerized decision support. Future research should explore which tools are most appropriate for pharmacists to use and how best to implement a practice change.

## Data Availability

At the time of ethical approval and participant consent no approval was obtained for public sharing of the data sets even in a de-identified form as such the data is not available.

## Abbreviations

REALM: Rapid Estimate of Adult Literacy in Medicine
TOFHLA: Test of Functional Health Literacy in Adults
LMPS: Lower Mainland Pharmacy Services
VIHA: Vancouver Island Health Authority
AHS: Alberta Health Services
UBC: University of British Columbia
NVS: Newest Vital Sign
